# Polyimide-Based Nanocomposites with Ultra-High Dielectric Breakdown Strength: A Review and New Record

**DOI:** 10.1021/acsaelm.5c01479

**Published:** 2025-10-21

**Authors:** Sombel Diaham, Imadeddine Benfridja, Tadhg Kennedy

**Affiliations:** † Université de Toulouse, Toulouse INP, CNRS, LAPLACE, Toulouse 31062, France; ‡ Department of Chemical Sciences, 8808 University of Limerick, Limerick V94 T9PX, Ireland; § Bernal Institute, University of Limerick, Limerick V94 T9PX, Ireland

**Keywords:** polyimide, nanocomposites, electrical insulation, high voltage, dielectric breakdown strength

## Abstract

A novel state-of-the-art record in the field of dielectric breakdown strength enhancement of polyimide-based nanocomposites is reported in this work. This achievement has been obtained through accurate optimization of the surface chemistry of silica (SiO_2_) nanoparticles. An efficient surface functionalization using 3-aminopropyltriethoxysilane (APTES) enabled the successful grafting of a single-layer ligand coverage onto SiO_2_ and to reach an optimal colloidal stability, promoting their homogeneous dispersion within the PI matrix. APTES-functionalized PI/SiO_2_ nanocomposite films exhibit significant improvements of the electrical insulation properties with lower permittivity, dielectric loss, and conductivity under high electric field, all related to more efficient dipolar motion restrictions and charge trapping effects. This study demonstrates for the very first time the path to design revolutionary ultrahigh breakdown field strength properties in a polyimide-based nanocomposite with E_BD_ ∼ 1000 V/μm and an enhancement factor η_E_ ∼68% compared to pure PI. Our results present a methodology for significantly advancing the state-of-the-art, enabling polyimide-based nanocomposite films to unlock future high-voltage applications, such as integrated insulation and capacitive energy storage.

## Graphical abstract

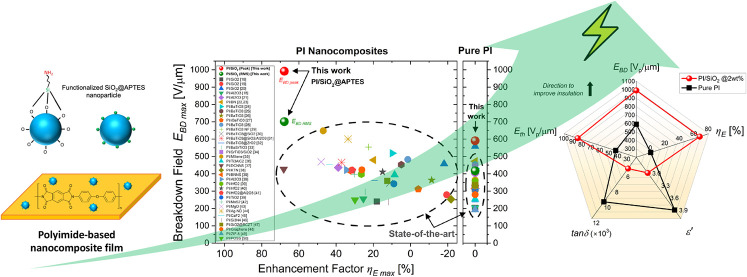

## 1 Introduction

The electronic and power semiconductor industry is expected to continue to grow significantly over the coming decades, strongly driven by the electrification of transportation (EVs, aerospace) and the high-voltage power conversion needs that incorporate new wide bandgap device technologies.^1,2^ These open new opportunities for dielectrics and electrical insulating polymer materials for high-voltage electronic applications such as thin-film capacitors,^3–5^ gate dielectrics,^6^ galvanic isolation,^7–9^ or semiconductor passivation,^10^ where insulation requirement is critical for system reliability. Since their discovery in the mid-1950s, polyimides (PIs) have emerged as key advanced materials for designing high-performance and reliable electronic devices and power systems from low to high voltage due to their excellent electrical, thermal, and mechanical properties and ease of processing.^11^ PIs are nowadays very suitable when considerations such as high power density, integration, high temperature, energy storage, high-voltage insulation or flexibility are required in order to meet the growing needs of global electrical energy consumption.^12^ One of the most desirable properties of PI films is the dielectric strength, which may be evaluated as either the short-term breakdown field strength (*E*_*BD*_) or the time-dependent breakdown voltage (TDDB). The primary design restriction for high-voltage systems is the intrinsic dielectric breakdown feature *E*_*BD*_, which predominates the design guidelines of dielectric materials.^8,13–15^ However, the increasing demand for more efficient electrical systems is driving the need for higher operating voltages, thinner insulation, and higher operating temperatures. These new specifications impose strict requirements for dielectric strength and temperature ratings for polyimide-based films. In order to fulfill such needs, several studies have been directed toward the development of advanced polymer-based nanocomposite materials dielectrically enhanced by the incorporation of various types of nanoparticles.^16,17^ In recent years, polyimide-based nanocomposite films have attracted a lot of research attention as a means to enhance the dielectric properties of PIs, with the objective of increasing their breakdown field *E*_*BD*_ for insulation and/or modifying their permittivity *ε*_*r*_ for capacitive energy storage.^18–50^ This was mainly achieved by nanostructuring the PI bulk and its interfaces with functionalized nanofillers of various types. Figure 1 compiles a large data collection of dielectric strength *E*_*BD*_ and breakdown enhancement factor η_*E*_ identified in various types of polyimide-based nanocomposites extracted from the 32 main reference papers in the literature. The data are replotted here as a function of the filler content. As breakdown varies with geometrical and environmental conditions, film thickness and measurement temperature are also indicated in the caption when they were available from the literature.

**Figure 1:**
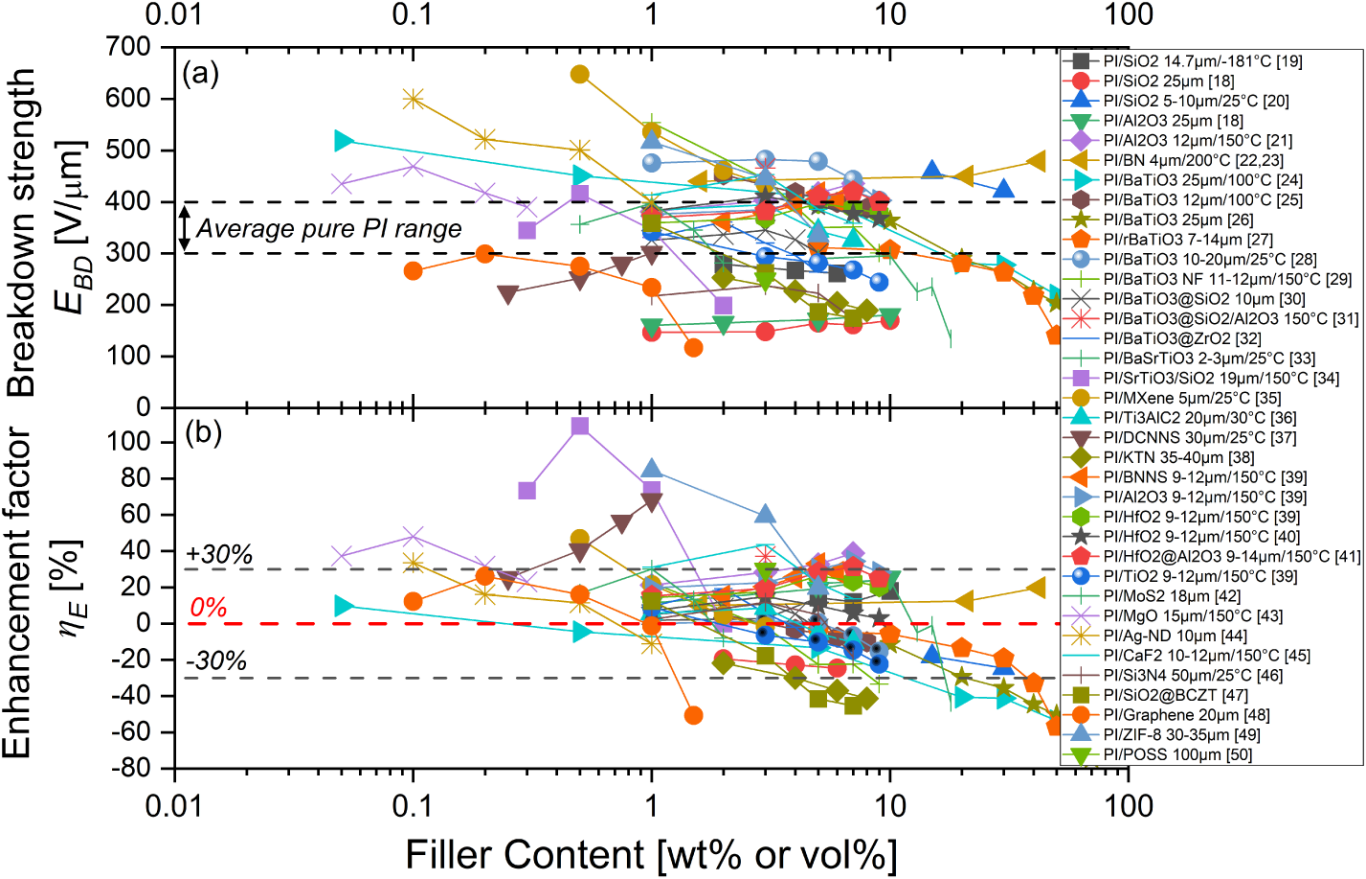
(a) Literature review of the dielectric breakdown strength *E*_*BD*_ of polyimide-based nanocomposites as a function of the filler content with different nanoparticles, taken from refs 18–50 . Film thickness and measurement temperature are indicated in the caption when available from the literature. The dashed line zone represents the typical mean breakdown field range for pure PI films. (b) The breakdown enhancement factor η_*E*_ corresponds to the normalized breakdown field with respect to that of the PI matrix obtained in each study. The dashed lines correspond to the typical improvement or deterioration ranges (±30%) observed in most of these studies.

First, one can observe the large discrepancy of the breakdown field, *E*_*BD*_, values across those studies that can depend on the nanofiller nature, size, shape, aggregate size and likely the optimization of the elaboration process (see Figure 1a). Although the breakdown performances have been notably improved over the last 10 years, such differences still highlight the difficulties encountered today in achieving predictive and systematic high dielectric strength in PI nanocomposite films. Second, it should also be noted that the breakdown field enhancement factor, η_*E*_, varies considerably from study to study (see Figure 1b). Overall, most of these studies have reported either an undesired reduction or a minor enhancement of the dielectric strength contained between η_*E*_ ∼ ± 30% for PI nanocomposites compared with the pure PI matrix. Only very few studies exhibit a large enhancement factor of η_*E*_ > + 30%. In some of them, however, it corresponded to a normalization with respect to an initial breakdown field of pure PI films lower than the typical *E*_*BD*_ average range and thus artificially increasing η_*E*_. More generally, it appears not trivial to enhance the breakdown field of pure PI films, particularly when they already start from high values of *E*_*BD*_ ∼ 400 V/μm, in order to reach ultrahigh *E*_*BD*_ > 600 V/μm for PI nanocomposite films requiring a significant enhancement factor η_*E*_ > 50%. It is worth noting that the highest intrinsic *E*_*BD*_ values ever obtained for pure PI films (5 and 12.5 μm) under AC and DC fields were recently reported as high as 505 V_rms_/μm and 705 V/μm, respectively.^8^ Until now, the cutting-edge state-of-the-art of dielectric breakdown strength for PI nanocomposite films was capped at *E*_*BD*_ = 648 V/μm and η_*E*_=+46.9% recently reported by Yu et al. in very singular PI/MXene nanocomposites tested in peak electric field conditions for 5 μm thick films at room temperature.^35^ The reason for such improvements was explained by an efficient electron capture by the MXene nanolayers (from a very low filler content 0.5 wt %), mitigating leakage currents and increasing the breakdown field. For other types of nanoparticles that are using conventional blending methods, and even if improvements are sometimes significant, PI-based nanocomposites are flattening at *E*_*BD*_ ≤ 600 V/μm, as reported by Xing et al. for Ag nanodots,^44^ or even below *E*_*BD*_ < 550 V/μm, as largely documented by Ru et al. on BaTiO_3_, by Ai et al. on TiO_2_, HfO_2_, Al_2_O_3_ and BNNS, and also by others on BN, MgO, and CaF_2_.^21–25,29,31,39–41,45^

Nowadays, it is broadly acknowledged that the enhancement of the dielectric properties of polymer-based nanocomposites is strongly related to the quality of the interface between the polymeric chains and the nanoparticles but also of the interphase bonded region which is inversely proportional to the nanofiller size.^17,51^ It is now widely documented that the breakdown strength maximization in polymer-based nanocomposites is closely correlated to the quality of nanofiller dispersion: meaning (*i*) mitigating the agglomeration of nanoparticles together and also (*ii*) enhancing the dispersion throughout the whole polymer matrix thus decreasing the nearest cluster distance between nanoparticles.^52^ One of the key routes for improving the nanoparticle dispersion is to functionalize their surface by ligand engineering with coupling chemical treatments that will ensure a good disaggregation of the fillers from each other.

Small electropositive ligands (e.g., silane or phosphonate coupling agents) are usually used to improve the enthalpic compatibility between nanoparticles and polymer chains.^53,54^ However, an optimal ligand surface design is required to maximize the dispersibility of nanoparticles.^55^ Ideally, a monolayer of ligand is optimal to enhance the filler–polymer interface and to investigate the role of this interface on mitigating leakage current and dielectric loss and to provide for higher breakdown strength.^54^ Contrary to other types of nanocomposites,^56^ polyimide-based nanocomposites are still suffering from a lack of investigation on the filler–polymer interface design, which is crucial for optimizing charge trapping effects and mitigating electrical degradation under high electric fields.

This paper investigates the effect of surface functionalization of silica (SiO_2_) nanoparticles with 3-aminopropyltriethoxysilane, a silane-based ligand, on the properties of a polyimide/SiO_2_ polymer nanocomposite. It reports on the optimization of the dispersibility of the nanofiller in a polyimide-based resin when the ligand is accurately grafted to their surface. It evaluates the effects of varying the filler content from the very low concentrations on a broad set of electrical properties of PI/SiO_2_ nanocomposites including the dielectric strength. Our results particularly highlight the key role of the filler dispersion optimization in order to reach, here for the very first time, what we call the *ultrahigh breakdown field strength* of polyimide-based nanocomposite films under AC peak field conditions with a world record established as high as *E*_*BD*_ ∼ 1000 V/μm.

## 2 Experimental Part

### 2.1 Materials

The SiO_2_ nanoparticles were purchased from Sigma-Aldrich with an average diameter of 18 nm. The surface modification of the SiO_2_ nanoparticles was carried out using 3-aminopropyltriethoxysilane (APTES, C_9_H_23_NO_3_Si) coupling agent as the ligand, also purchased from Sigma-Aldrich. All the other chemicals were of reagent grade and were used without further purification.

The polyimide matrix used in this study was prepared from a liquid polyamic acid solution (PAA), where a 4,4’-oxidianiline (ODA) and a pyromellitic dianhydride (PMDA) both dissolved in an *N*-methyl-2-pyrrolidone (NMP) polar solvent. This PAA is the precursor of the final poly­(4,4’-oxidiphenylene pyromellitimide) (PMDA-ODA) polyimide once the imidization reaction is complete. The deposition and imidization curing process steps were previously optimized on pure polyimide in order to maximize its dielectric breakdown properties. All the details were previously reported.^57^

### 2.2 Surface Modification of SiO_2_ Nanoparticles with APTES Ligand

The surface functionalization of SiO_2_ nanoparticles were achieved by first mixing 200 mg of SiO_2_ in 100 mL of ethanol in a 250 mL beaker and by stirring with a magnetic stirring plate for 15 min. Then, a proper volume of APTES from 2 to 10 μL was added to different dispersed ethanol/SiO_2_ solutions ranging from 1 to 5 wt % of APTES in steps of 1 wt %. The different ethanol/SiO_2_/APTES mixtures were stirred for 1 h at room temperature, before being further dispersed using a sonication bath for 3 h, and let react for 24 h to accomplish the hydrolysis and condensation of the ligand onto the SiO_2_ surface, as depicted in Figure 2a. Surface APTES-functionalized SiO_2_ (labeled SiO_2_@APTES) was then recovered by centrifugation and subsequently washed with ethanol twice followed by vacuum drying at 100 °C for 2 h. For the control, untreated SiO_2_ nanoparticles were first stirred and sonicated in ethanol without APTES in the same way, followed by direct centrifugation, before being vacuum-dried in the same conditions.

**Figure 2:**
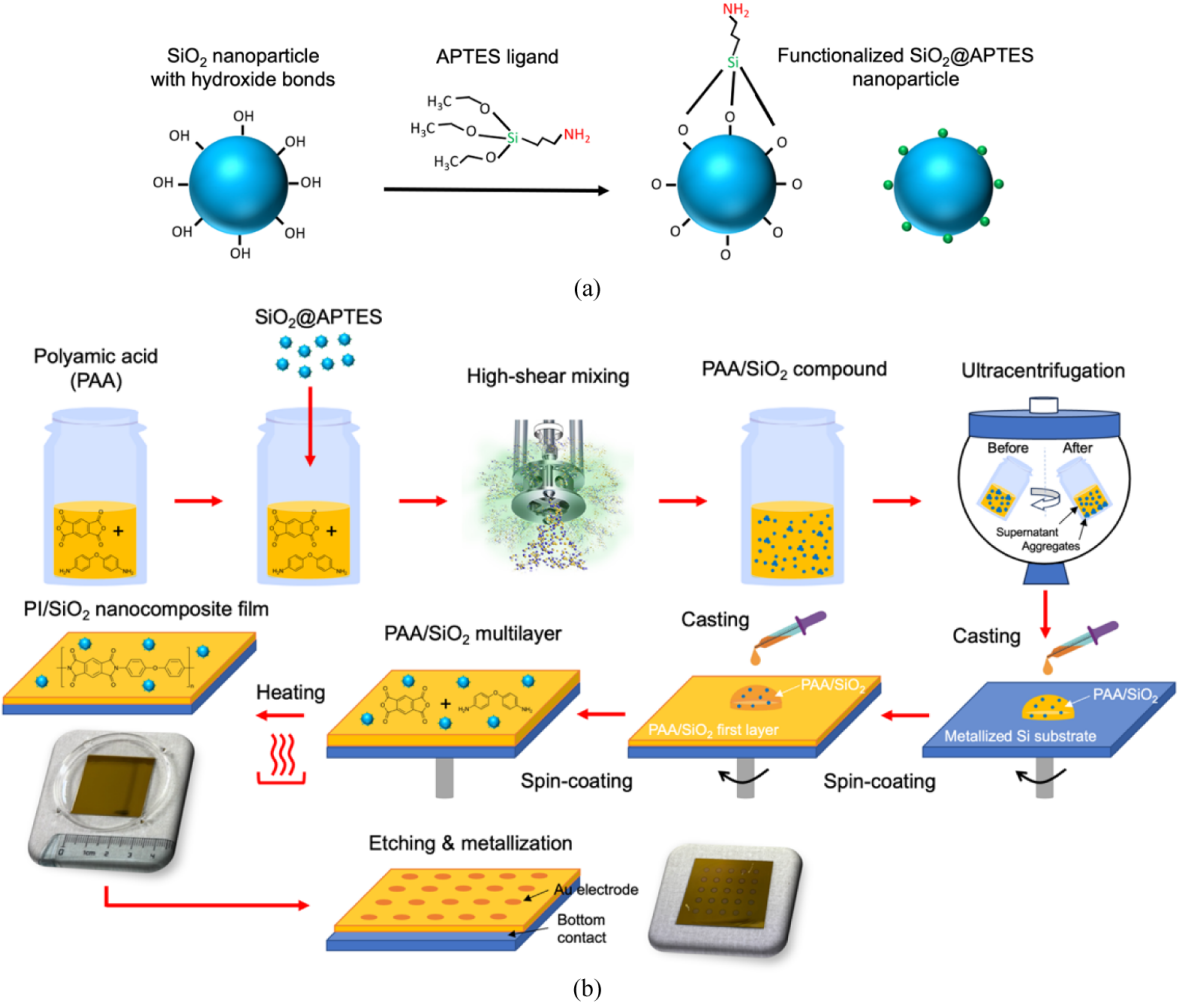
(a) Reactional scheme of the SiO_2_ nanoparticle surface functionalization with the APTES ligand. (b) Fabrication process of the multilayer PI/SiO_2_ nanocomposite films.

### 2.3 Fabrication of PI/SiO_2_ Nanocomposite Films

Both untreated SiO_2_ and SiO_2_@APTES nanoparticles were added to the PAA solution with different filler contents ranging from 0.1 to 2 wt % and then dispersed by high-shear mixing at 5,000 rpm for 90 min while maintaining the solution temperature close to ambient conditions using a surrounding cooling bath. Subsequently, to remove as best as possible residual large aggregates, a centrifugal decantation by ultracentrifugation was used to reduce them in size and density. To achieve this, the PAA/SiO_2_ compound solutions were placed in tubes before being subjected to a centrifugal force of 21,000 G (14,800 rpm) for 5 min. The supernatant, containing mainly the nanodispersed phase, was then recovered and used to prepare the PI/SiO_2_ nanocomposites. To characterize the dielectric properties, the resulting SiO_2_/PAA mixtures were spin-coated onto square-diced Si wafer substrates coated with a blanket metal layer. The spin speed was adjusted to yield PI/SiO_2_ nanocomposite films with a 5 μm thick single-layer process. Two layers were consecutively cast to double the film thickness. Thermal curing in an oven under nitrogen atmosphere enabled completion of the PI chemical reaction. The postcure final film thickness was 10 ± 0.4 μm for the whole filler concentration range. The electrical test structures were completed by patterning gold circular electrodes of 1 mm in diameter by Ar-plasma sputtering with a thickness of 50 nm. All the process flow is depicted in Figure 2b.

### 2.4 Characterization

Attenuated total reflectance Fourier transform infrared (ATR-FTIR) spectroscopy was performed on the untreated and SiO_2_@APTES nanoparticles using a Cary 630 FTIR spectrometer. An average of 128 scans was collected for each measurement in the wavenumber range from 600 to 4000 cm^–1^. X-ray photoelectron spectroscopy (XPS) was used to detect the presence of surface elements and the presence of Si, O, *N*, and C bonds on the modified SiO_2_@APTES nanofillers. The zeta potential ζ of the untreated SiO_2_ and SiO_2_@APTES nanoparticles in ethanol were measured using a Zetasizer Nano ZS instrument (Malvern, England). The nanofiller dispersion in the nanocomposite films was characterized by both scanning electron microscopy (SEM) and transmission electron microscopy (TEM) using Hitachi models (Tokyo, Japan) to observe the morphology of untreated SiO_2_, modified SiO_2_, and the various PI/SiO_2_ nanocomposites. Cross-sectioning of the films was obtained by either focused ion beam (FIB) etching or cryogenic cleaving. Dispersion was also assessed by atomic force microscopy (AFM) performed using a Bruker Multimode 8 apparatus. Prior to all the electrical tests, the samples were dried in an oven at 150 °C for 2 days to get rid of any moisture. Permittivity, dielectric loss, and alternating conductivity were all measured by high-voltage broadband dielectric spectroscopy (HVBDS) at room temperature and between 1 and 1400 V_rms_ applied voltage range using a Novocontrol Alpha-A HVB4000 spectrometer (Germany). AC breakdown under a 50 Hz sinewave ramped voltage was achieved (∼1 kV/s) by contacting electrodes using a probe-station to a high-voltage amplifier, as shown earlier,^8^ with details in Figure S1. A total of 20 samples failed per type of nanocomposites and then statistically analyzed with the Weibull distribution. Several sample lots (at least three) were tested per condition and have shown good reproducibility. All of the breakdown tests were carried out at 25 °C by immersing the samples in insulating oil (Galden HT270) to prevent flashover. During the tests up to failure, both the applied voltage and related current waveforms were recorded on an oscilloscope through an HV probe and a high-frequency current transformer sensor (HFCT), respectively.

## 3 Results and Discussion

### 3.1 SiO_2_ Surface Functionalization with APTES Ligand

Untreated and APTES-functionalized SiO_2_ nanoparticles were examined by using ATR-FTIR to detect the characteristic covalent chemical bonds of the functionalization ligand, as presented in Figure 3. The transmittance bands located at 810 cm^–1^ and 1112 cm^–1^ in the FTIR spectrum of untreated SiO_2_ nanoparticles are attributed to the Si–O stretching mode and the siloxane vibrations of (SiO)_*n*_ groups, respectively, as referenced elsewhere.^58,59^ The shoulder located at 3445 cm^–1^ is assigned to the O–H stretching band of the surface of silanol groups. APTES presents its own Si–O bonds but also −NH_2_, −CH_2_, and −CH_3_ vibration modes all present in the molecule backbone, as previously identified.^60–62^ Besides, the appearance of a band in the regions of 1400 cm^–1^ and 1468 cm^–1^ can be attributed to N–H vibration, and the bands in the region of 2800–2980 cm^–1^ are assigned to CH_2_ and CH_3_ stretching.^61,62^

**Figure 3:**
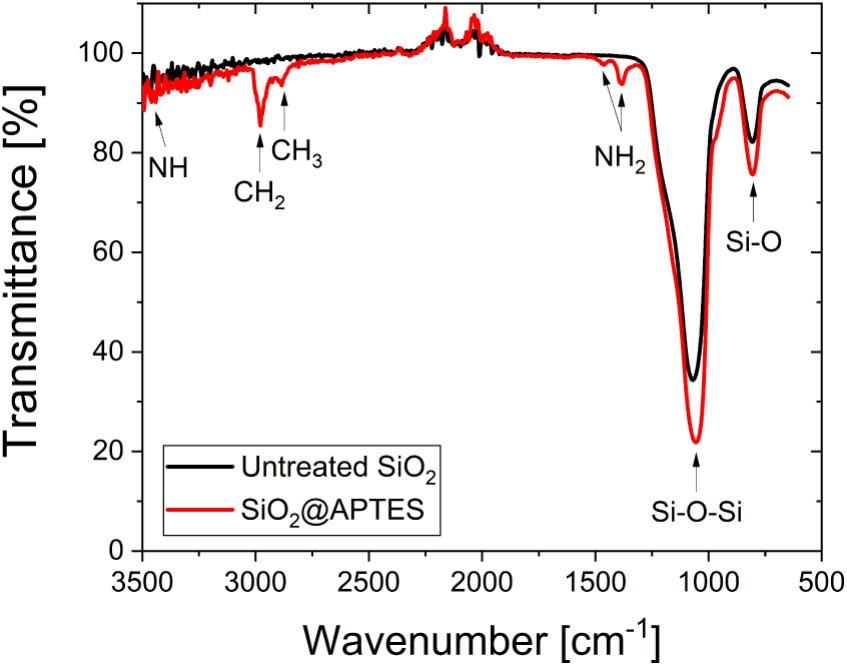
FTIR spectra for untreated SiO_2_ and SiO_2_@APTES-functionalized nanoparticles.

To further confirm the functionalization of the nanoparticles, the extreme surface composition of untreated and APTES-functionalized SiO_2_ was investigated by XPS. Broad scan XPS spectra of untreated SiO_2_ and APTES-functionalized SiO_2_ for ligand contents of 1 and 5 wt % are presented in Figure 4. The untreated SiO_2_ spectrum exhibits three characteristic peaks for binding energy at 106, 155, and 532 eV corresponding to Si 2p and O 1s elements, respectively. However, the XPS spectra of the APTES-functionalized SiO_2_ nanoparticles shows two additional peaks located at 400 and 285 eV and that correspond to the N 1s and C 1s peaks, respectively, derived from C_9_H_23_NO_3_Si during the functionalization process.^61–66^ The XPS spectrum in Figure 4b intensifies the N 1s region and shows its deconvolution. Thus, the N 1s peak is made up of two subpeaks: the main one located at 399.8 eV, which is related to the amine group bounded to carbon C–NH_2_, and a second, smaller one located at 402 eV that could be attributed to protonated amine groups −NH_3_^+^ probably due to the direct acid–base interaction of the amine with the carboxylic and/or OH groups.^63,65^ A deconvolution study of the C 1s peak (Figure 4c) shows four components. A first subpeak at a binding energy of 284.8 eV is attributed to the C–C and C–H groups,^62,65^ and a second subpeak at 285.6 eV is related to the C–N and C–O bonds. A third component at 286.8 eV corresponds to the C–O and C = O groups, and a fourth component observed at 288.4 eV is assigned to the H–O–C = O carboxylic groups.^62,65^

**Figure 4:**
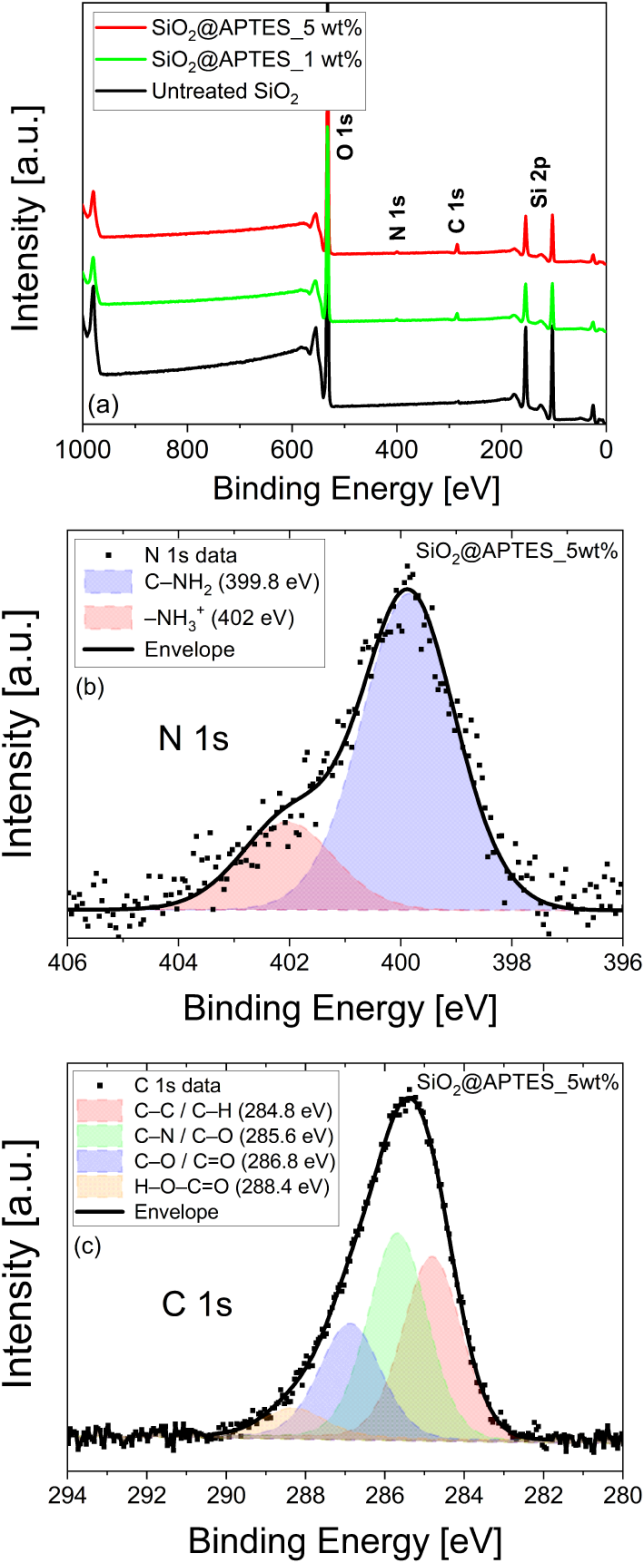
(a) XPS survey for untreated SiO_2_ and for SiO_2_@APTES 1 and 5 wt % functionalized nanoparticles. (b) N 1s peak deconvolution for SiO_2_@APTES 5 wt %. (c) C 1s peak deconvolution for SiO_2_@APTES 5 wt %.

The APTES functionalization induces a significant increase in the intensity of the characteristic peaks for N 1s and C 1s, both strongly related to the functional groups forming part of the APTES ligand chemical structure. FTIR and XPS signal increments, observed for both covalent bonds or elements containing nitrogen and carbon, thereby confirm the successful grafting of APTES ligand molecules onto the surface of the SiO_2_ nanoparticles.

The effects of the surface silanization of SiO_2_ nanoparticles were first examined by TEM to determine the average shape and dispersion quality of the nanofillers. Figure 5 depicts TEM images of untreated SiO_2_ and APTES-functionalized SiO_2_ nanoparticle suspensions in ethanol at various scales. Figure 5a,c clearly shows larger nanoparticle agglomeration for untreated SiO_2_, whereas Figure 5b,d shows that their dispersion after APTES functionalization is greatly improved. Furthermore, it was found that the surface modification significantly enhances the nanoparticle average size, which was smaller than that of untreated SiO_2_. However, TEM images of both treated and untreated nanoparticles reveal also a significant amount of size inhomogeneity. The untreated SiO_2_ shows aggregate diameters ranging between 40 and 60 nm compared to 10 to 30 nm for functionalized SiO_2_ nanoparticle aggregates, with pseudospherical shape for both types. Therefore, the APTES treatment effectively helps to reach the expected SiO_2_ average size close to 18–20 nm by limiting the ability of the nanoparticles to aggregate.

**Figure 5:**
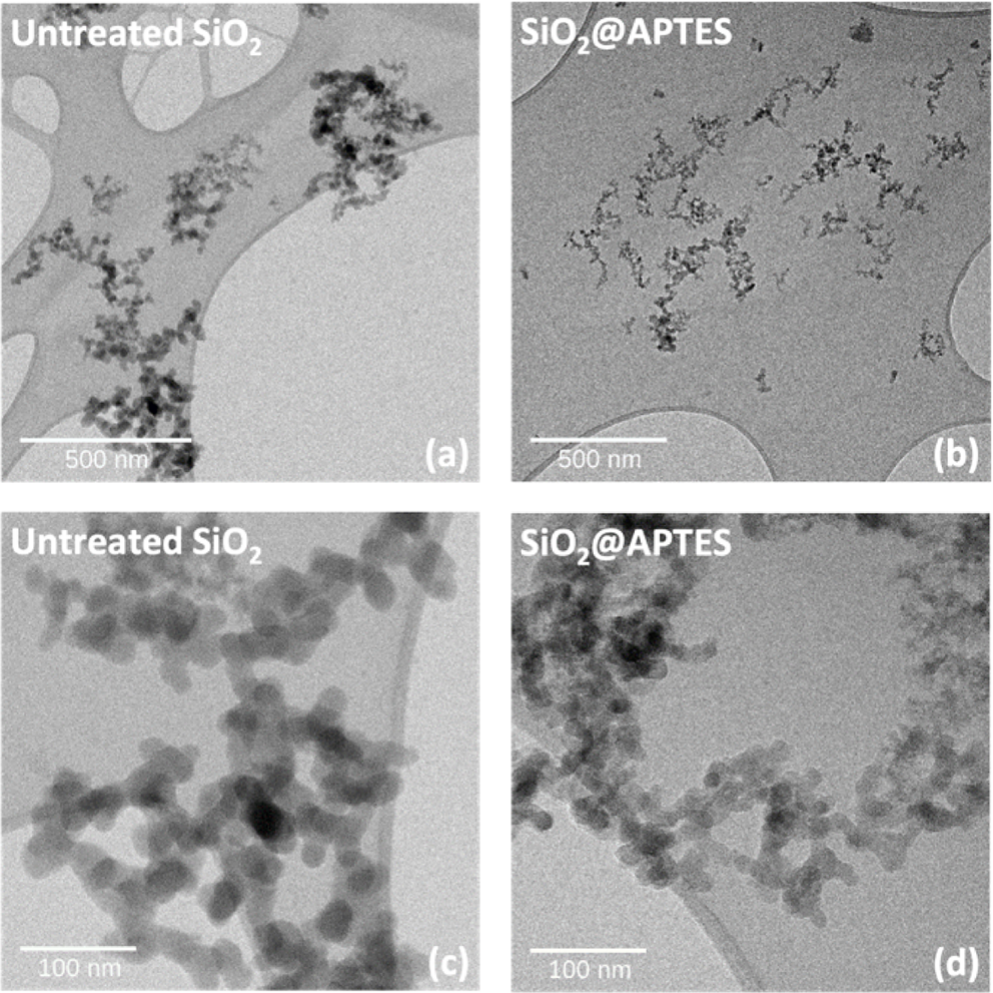
TEM images of the untreated SiO_2_ (a, c) and 5 wt % APTES-functionalized SiO_2_ nanoparticles (b, d) dispersed in ethanol. Scale bars are 500 and 100 nm.

To evaluate the colloid stability of the modified SiO_2_, which further assesses the grafting efficiency of the ligand, ζ-potential was employed to measure the surface potential of the nanoparticles in initially neutral ethanol. An average of 3 tests of 20 measurements each was performed. The results, presented in Figure 6, exhibit monotonic behavior with a progressive increase of ζ with an increase in the APTES concentration. The ζ-potential has been found to increase from a very low value at 0.74 ± 0.5 mV for the untreated SiO_2_ up to reaching high values at 36 ± 1.2 mV for functionalized nanoparticles with 5 wt % of APTES. A ζ-potential between 30 and 40 mV (either positive or negative) is considered a good indication of the nanoparticle stability according to the ASTM standard for the stability of colloidal suspensions.^67^ Such an improvement is due to the abundance of the protonated amine groups (−NH_3_^+^) on the surface of the APTES-functionalized SiO_2_, as confirmed by XPS (Figure 4), which results in an increase of the surface charging.^64,68,69^ Consequently, for APTES concentrations between 1 and 4 wt %, the protonic surface charge of the functionalized SiO_2_ increases rapidly and continuously. This emphasizes that more and more ligand groups are grafted onto the SiO_2_ nanoparticle surface. On the contrary, from 5 wt %, the ζ-potential tends to stabilize due to the partial shield of the APTES group with multilayer form.^70,71^ It is thereby acceptable to conclude that a complete monolayer of grafted ligand is obtained for a 5 wt % concentration of APTES considering the SiO_2_ nanoparticles studied here.

**Figure 6:**
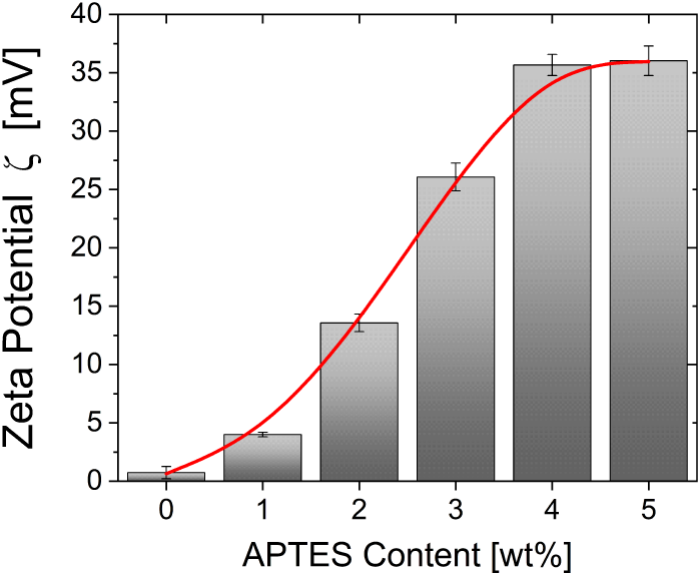
Zeta potential ζ of the SiO_2_ nanoparticles dispersed in ethanol as a function of the APTES content.

The above results from FTIR, XPS, TEM, and ζ-potential all suggest the successful functionalization of the SiO_2_ nanofillers.

### 3.2 SiO_2_ Dispersion in PI-Based Nanocomposite Films

Before the impact of nanostructuring on the electrical properties of polyimide nanocomposites was investigated, the effect of APTES functionalization was first analyzed in terms of nanoparticle dispersion quality within the PI matrix. For this purpose, untreated and 5 wt % APTES-functionalized SiO_2_ nanoparticles were dispersed in PI films, as they showed the highest ζ-potential value. Surface morphology and roughness of the samples were characterized by AFM in scanning the surface topography in tapping mode. AFM images with a scan size of 10 × 10 μm^2^ are presented in Figure 7 for both untreated and APTES-functionalized PI/SiO_2_ (2 wt %) nanocomposite films.

**Figure 7:**
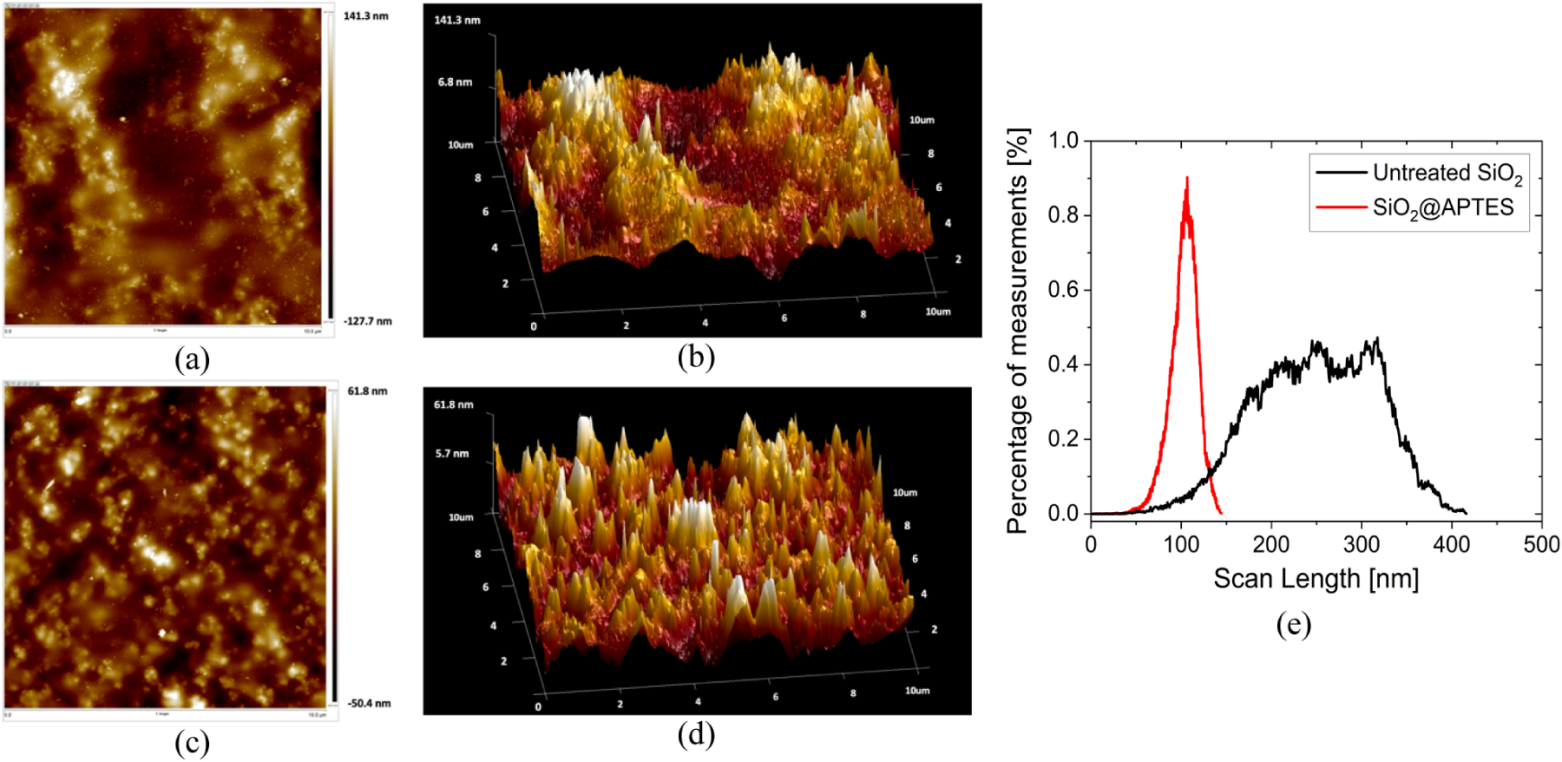
AFM surface topography of the untreated (a, b) and APTES-treated (c, d) PI/SiO_2_ nanocomposites (scan size of 100 μm^2^). Example of the typical aggregate size distribution comparison for each nanocomposite (e).

According to these, the topography image for the untreated PI/SiO_2_ nanocomposite presents a rough surface and the fillers appear to be stacked and agglomerated due to the van der Waals interparticles attraction (Figure 7a). However, the functionalized PI/SiO_2_ nanocomposite was less prone to aggregation and sedimentation. As a result of the electrostatic repulsion caused by the APTES grafting, it is clear that the nanoparticle dispersion within the PI matrix is significantly improved for functionalized SiO_2_ (Figure 7c). The use of root-mean-square values derived from the topography scans demonstrates the change in roughness more clearly. The [*R*_*q*_, *R*_*a*_, *R*_*max*_] parameters were calculated throughout each whole image and were equal to 38.1, 30.1, and 338 nm, respectively, for the untreated PI/SiO_2_ (Figure 7b), compared to 15.4, 11.9, and 145 nm for the functionalized nanocomposite (Figure 7d). In addition, the image surface area differences were of 4.86% and 1.19%, for those, respectively. This difference in the roughness between the two nanocomposites confirms a much higher dispersion quality of the functionalized SiO_2_ into the PI matrix, as already observed in other nanocomposites.^72^ Furthermore, from the surface topography shown in Figure 7b,d, representative aggregates have been scanned to determine their average diameters and they were around 350 and 100 nm for the untreated and functionalized PI/SiO_2_ nanocomposite films, respectively, as illustrated in the distribution diagrams in Figure 7e. To note that these sizes are to be cautiously considered as they are influenced by the location depth of the aggregates from the surface, as well as by the well-known interaction phenomenon caused by the AFM tip convolution effects.^73,74^

In order to obtain a more accurate in-depth view of the nanoparticle dispersion for each nanocomposite, the effect of the functionalization has been evaluated by cross-sectional SEM imaging. Figure 8a–c shows the SEM images after FIB cross-sectioning of the pure PI bulk, the untreated and the APTES-functionalized PI/SiO_2_ at 2 wt % filler content, respectively, and for different magnifications. In the micrographs, one can observe that the PI/SiO_2_@APTES nanocomposite film exhibits a better dispersion of the nanoparticles in bulk compared with the untreated ones. The functionalized nanocomposites are uniformly distributed and have smaller aggregates (in the range of 100 nm) that are more randomly distributed throughout the bulk.

**Figure 8:**
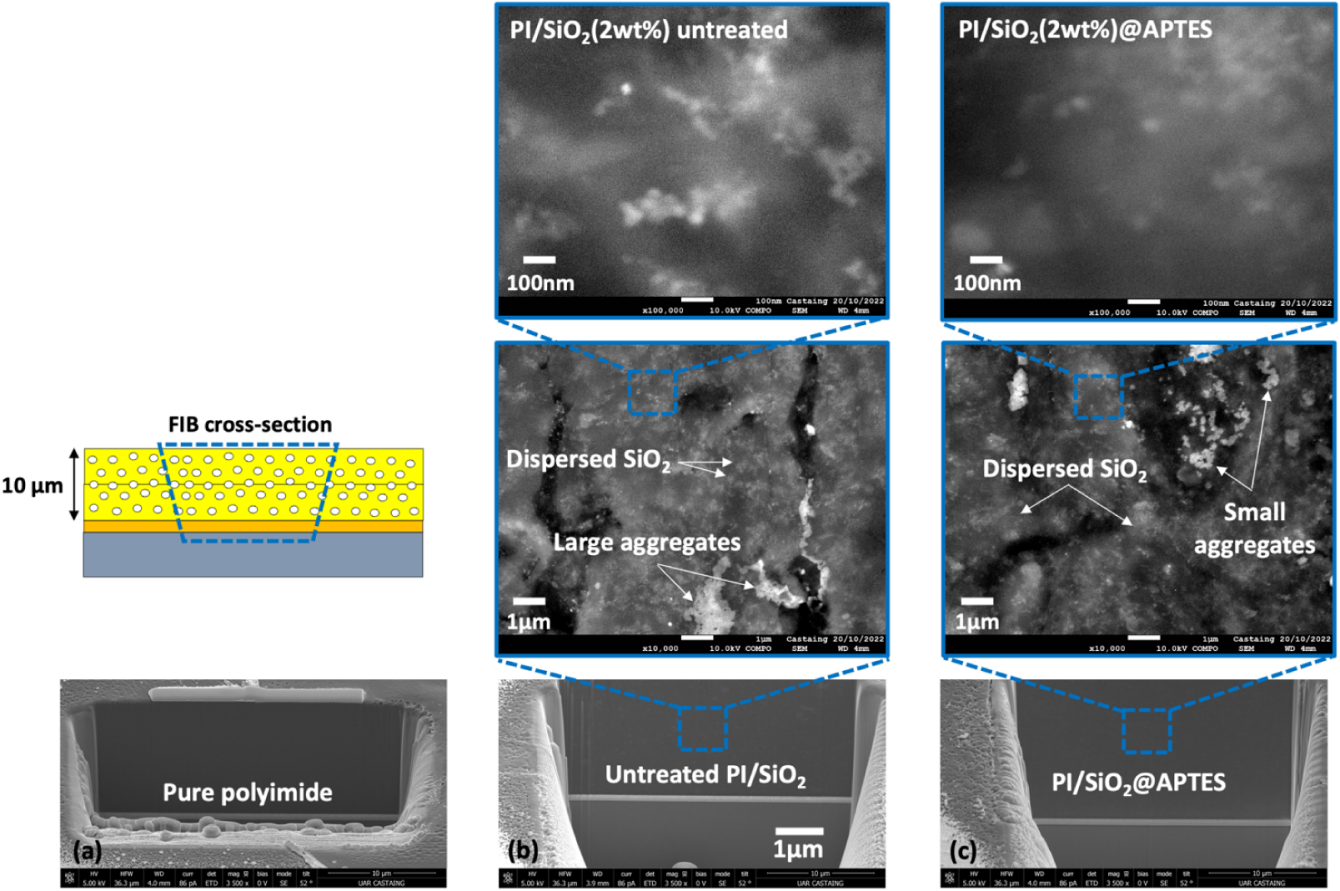
Cross-sectional FIB-SEM images with different magnifications in neat PI (a), untreated PI/SiO_2_ (2 wt %) (b), and functionalized PI/SiO_2_@APTES (2 wt %) (c).

Both AFM and SEM results confirm the good dispersion quality of the modified SiO_2_ in the PI nanocomposite films.

### 3.3 Dielectric Properties of PI/SiO_2_ Nanocomposite Films

For the rest of the study, only the effects of the optimal APTES-functionalized SiO_2_ nanofillers with a 5 wt % ligand surface treatment on the electrical properties are presented. For that purpose, a set of PI/SiO_2_@APTES nanocomposite films with different filler contents ranging from 0.1 up to 2 wt % have been prepared.

Figure 9 presents the dielectric properties changing at 50 Hz in the PI/SiO_2_ nanocomposite films as a function of the filler content. While the permittivity *ε’* and loss factor *tanδ* for the pure PI film are as high as 3.8 and ∼10^–2^, respectively, a significant decrease of both for all the PI/SiO_2_ nanocomposites tending to 2.9 and 5.5 × 10^–3^, respectively, is observed. As shown in Figure S2a, the permittivity of both pure PI and all the nanocomposite films gradually exhibits an expected decrease with increasing frequency. However, the dielectric polarization strength *Δε’*, which is mainly related to a dipolar orientation originating from short PI molecular chain segments, shows a larger reduction in the PI/SiO_2_ nanocomposites than in pure PI due to the influence of the nanofillers (see Figure S2b).

**Figure 9:**
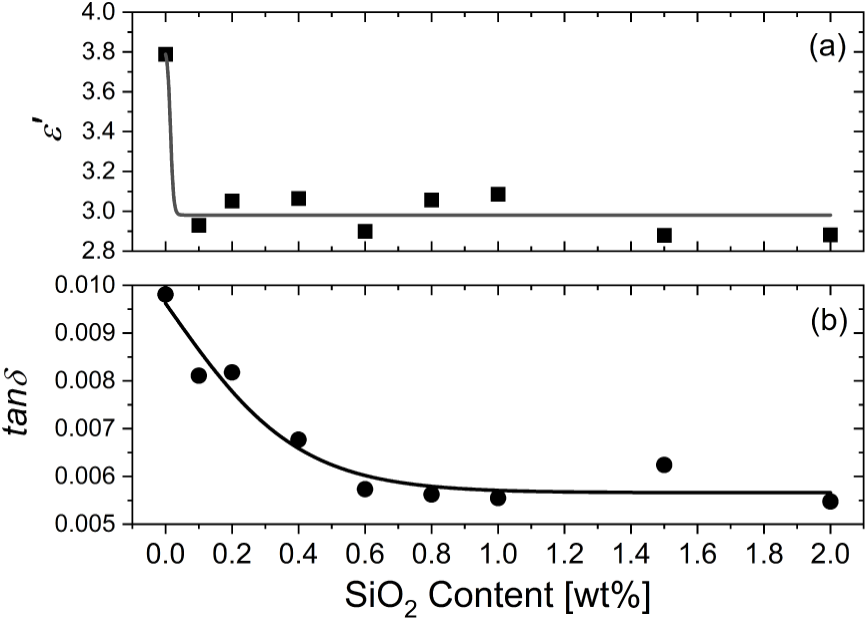
Permittivity (a) and dielectric loss factor (b) changing at 50 Hz and under 1 V in the PI/SiO_2_ nanocomposite films as a function of the SiO_2_@APTES filler content (measurement error: 1%).

The good dispersion of the nanoparticles in the polyimide films attenuates the molecular mobility of dipoles along the polyimide chains due to the greater confinement effects developed and restricting chain’s motions. As mentioned earlier, it was shown that the introduction of nanoparticles creates large interface areas with the matrix.^17,75^ The trap level and their distribution energy within the nanocomposite are influenced by both interface and interphase regions, which reduces the overall polarizability.^76^

Such an enhancement of the interphase region in the vicinity of SiO_2_ nanoparticles also has other positive electrical effects on the nanocomposite conduction properties. A first confirmation of the charge trapping improvement is given in Figure 10a, which plots the electric field dependence of the low-frequency AC conductivity (measured by HVBDS) in neat PI and PI/SiO_2_ nanocomposite films for the different filler concentrations. The polyimide matrix presents an AC field dependence of the conductivity that has been recently described.^77^ In such low-frequency AC high-field conditions, it was earlier reported that the field-dependent conductivity measured either in DC or AC are equivalent.^77^ Here, it shows a nonlinear behavior with a rapid raise of the low-frequency conductivity beyond a threshold field *E*_*th*_ ∼ 30 V_rms_/μm (∼42 V_p_/μm), which has been assigned to a Poole–Frenkel detrapping mechanism. On the other hand, the functionalized PI/SiO_2_ nanocomposite films show a much lower AC conductivity compared to that of neat polyimide with also a shift of the threshold field *E*_*th*_ ranging now from 50 V_rms_/μm to 60 V_rms_/μm (∼85 V_p_/μm) for filler contents from 0.1 to 2 wt %, respectively. As seen above for the dielectric properties, the significant doubling of the threshold field can be explained by a more efficient capturing of the charge carriers which is tuned in the nanoparticles’ interphase region and that reduces their mobility. The structuring of the PI matrix with SiO_2_ nanofillers offers the possibility to further delay the onset of the Poole–Frenkel detrapping process to much higher electric field values and to extend the potential working electric field range for all the nanocomposites. Based on our recent and earlier work on the trapping characterization evaluated by thermally stimulated depolarization current techniques (TSDC), one can say that polyimide nanocomposite films exhibit a significant trap density and trap depth modification with positive impacts on the space charge mitigation.^78^ This behavior has been imputed to the introduction of deep traps within the energy band structure, which act as injection barrier and/or effective mobility mitigators, ensuring a limited charge density inside the material.

**Figure 10:**
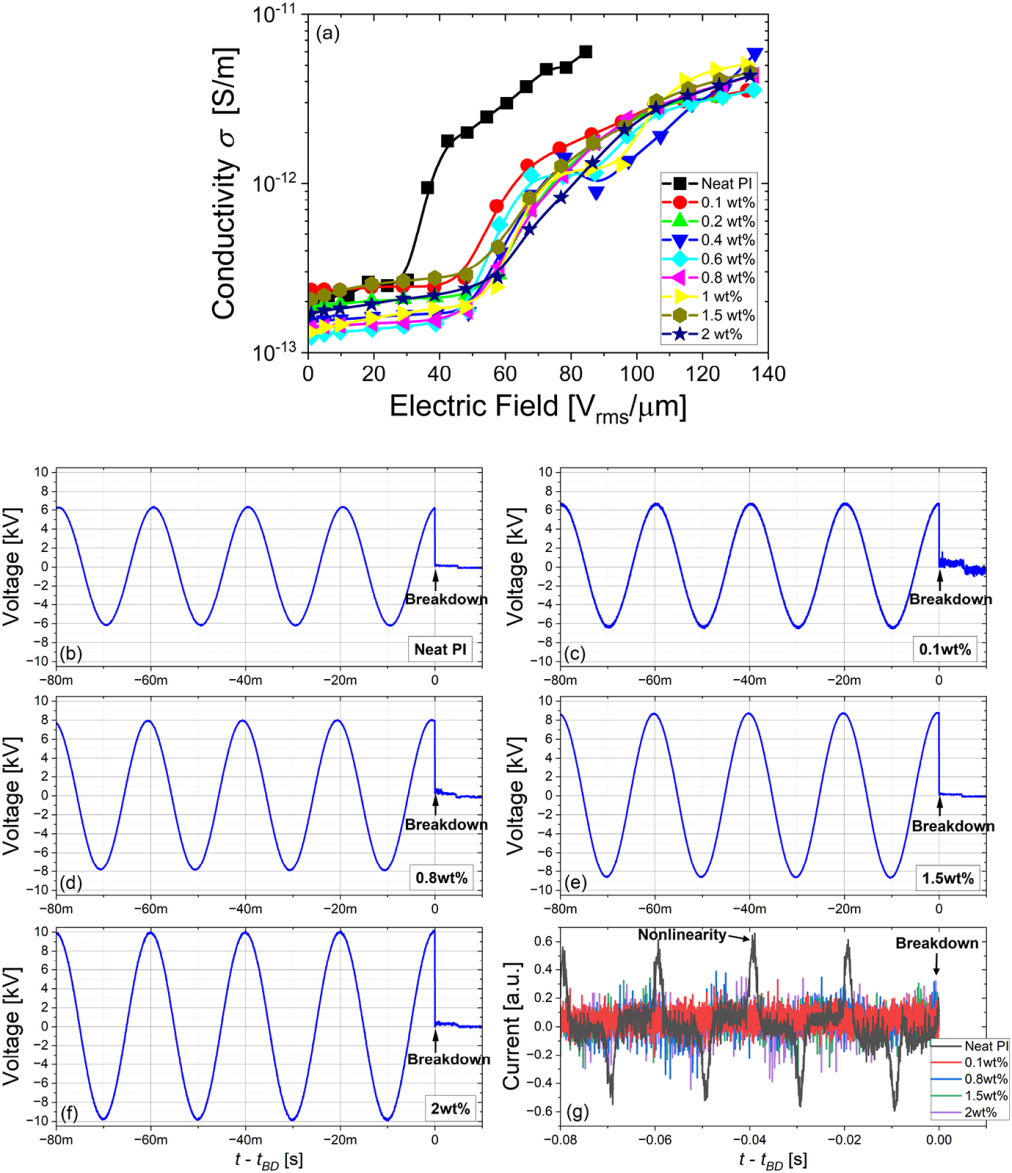
E-field dependence of the conductivity at 0.1 Hz in neat PI and PI/SiO_2_ nanocomposite films for different SiO_2_@APTES filler contents (a). Typical applied AC voltage waveform at 50 Hz in the last four periods before breakdown for different PI/SiO_2_@APTES nanocomposites (b–f) and their related prebreakdown current waveforms (g).

Figure 10b–g emphasizes the impact of applying extremely high AC voltages to the different nanocomposite films and their subsequent consequences on the current probed by HFCT at 50 Hz. The typical applied AC voltage waveforms at 50 Hz probed up to the very last four periods before breakdown are shown. The prebreakdown current in the PI matrix exhibits a further nonlinear behavior with peak maxima in-phase with the applied voltage (Figure 10g). This nonlinear conduction occurrence has been attributed to a thermal imbalance in the bulk leading to the breakdown occurrence.^8^ On the contrary, all the PI/SiO_2_ nanocomposites do not present such nonlinearity close to breakdown (or at least not detected by the HFCT sensitivity). Therefore, the nanocomposite films control their current more efficiently, allowing them to withstand much higher applied voltages than the pure PI. As an example, the typical peak breakdown voltage increases from 6 kV up to 10 kV for pure PI and PI/SiO_2_ (2 wt %), respectively.

From Figure 10b–f, the equivalent waveform of the AC electric field bias before breakdown was calculated for all the nanocomposites, and the results of the average material for each case are replotted in Figure 11a. Typically not shown in the literature, these types of AC electric field prebreakdown waveforms for each average PI nanocomposite film clearly highlight the high-field insulation enhancement that supports further the Weibull distribution and gives credit to the extremely high breakdown field values reported here. One can observe that the maximum electric field that the PI/SiO_2_ nanocomposite is capable of withstanding is dramatically and consistently increased with increasing filler content. This demonstrates the enhanced electrical performance of these nanocomposite films when adding a very low amount of nanofillers with a high-quality level of dispersion within the PI matrix. As failure is a stochastic phenomenon, the dielectric breakdown strength for all the PI/SiO_2_ nanocomposite films has been analyzed using the two-parameter Weibull statistical distribution law, as given by

1 $$P\left(E\right)=1-\exp\left[{\left(-\frac{E}{E_{\mathrm{BD}}}\right)}^{\beta }\right]$$

where *P*(*E*) is the cumulative breakdown probability, *E* is the measured electric field strength, *E*_*BD*_ is the Weibull breakdown strength at *P*(*E*)=63.2%, and β is the shape parameter evaluating the scatter of the 20 tested data.^79^

**Figure 11:**
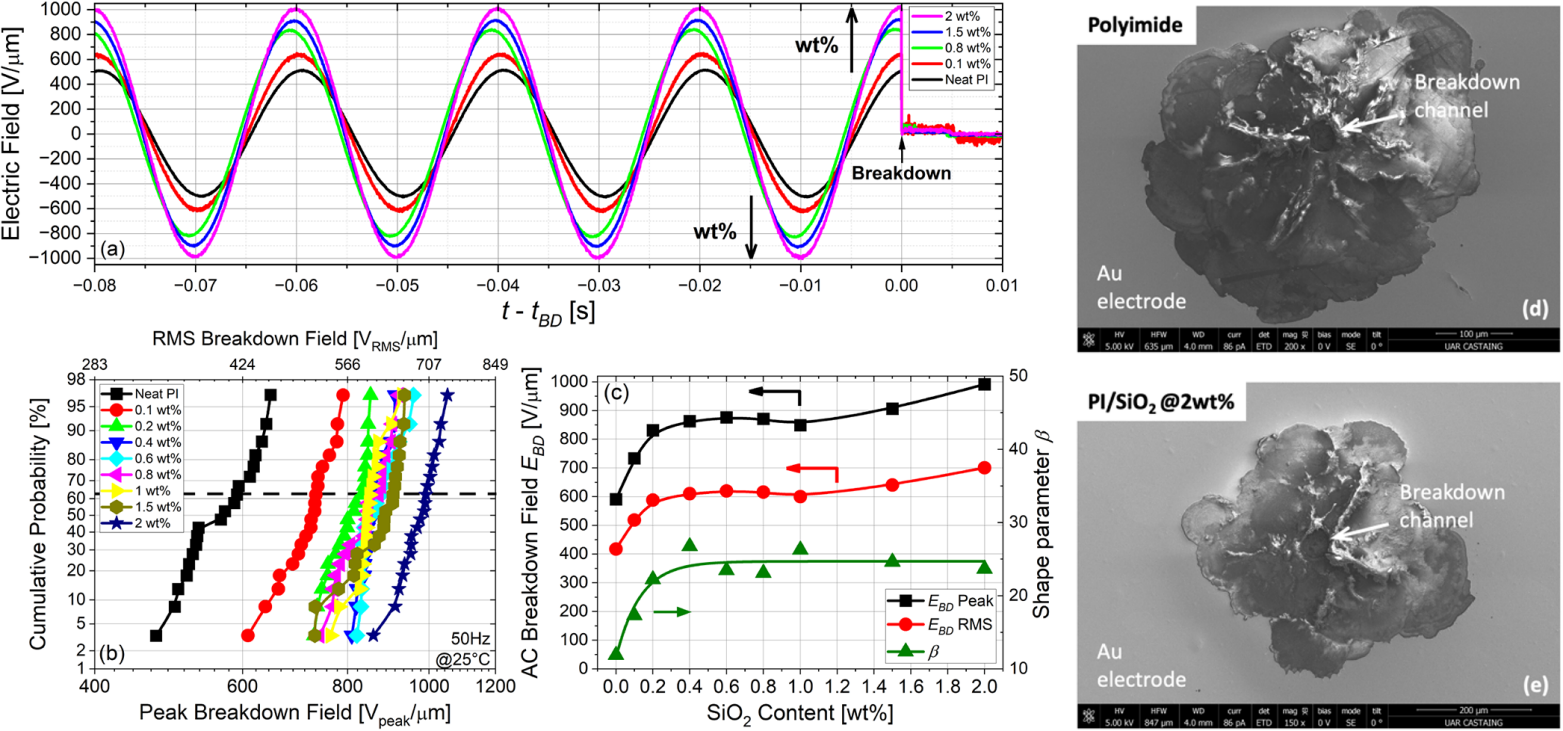
Typical applied AC electric field waveforms at 50 Hz in the last four periods before breakdown for different PI/SiO_2_@APTES nanocomposites (a). Weibull cumulative probability of failure vs both the AC peak and RMS breakdown fields for the different SiO_2_ filler contents (b). AC dielectric breakdown field (63.2%) in peak and RMS values and shape parameter vs the SiO_2_ filler content (c). Top-view SEM images of the breakdown channels for pure PI (d) and PI/SiO_2_@APTES 2 wt % nanocomposite films (e).

Figure 11b shows the Weibull cumulative probability of failure for the different PI/SiO_2_ nanocomposite films as a function of the AC breakdown field at 50 Hz. Moreover, Figure 11c replots the derived breakdown field strength *E*_*BD*_ (in peak and root-mean-square values) and the β-parameter versus the filler content. The pure PI film performs well, starting already high with values of *E*_*BD*_ = 590.6 V_p_/μm (∼418 V_rms_/μm). However, with as little as 0.1 wt % of SiO_2_ nanoparticles dispersed, *E*_*BD*_ increases significantly to 733.4 V_p_/μm. More interestingly, for higher SiO_2_ filler concentrations, the breakdown field of the nanocomposite films continues increasing monotonically to reach *E*_*BD*_ = 991.4 V_p_/μm (∼701 V_rms_/μm) at 2 wt % of SiO_2_ nanoparticles. This corresponds to the highest values ever reported for polyimide-based nanocomposite materials.^8,35^ Such enhancement of the breakdown field can be related to an increase in the deep trap density in the interphase regions around the SiO_2_ nanoparticles, and attenuating the mobile charge carriers that lead to failure.^23,80^ Meanwhile, the β-parameter is improved with the filler content with values increasing progressively from 12 to 25, which emphasizes the narrowing of the data discrepancy around the *E*_*BD*_ mean value. Figure 11d–e shows typical SEM images of the postbreakdown channels for pure PI and 2 wt % PI/SiO_2_ nanocomposite films. The final failure mechanism remains unchanged, as revealed here by the same kind of breakdown paths located underneath the gold electrode in both cases. This confirms the intrinsic breakdown feature for both films (as described previously).^8^ Therefore, nanostructuring intrinsically affects the dielectric properties in a positive way.

### 3.4 Breakdown Field Comparison with the State-of-the-Art

In order to compare the results obtained here in the case of PI/SiO_2_ nanocomposite films with the state-of-the-art PI-based nanocomposite, the breakdown field strength has also to be analyzed in terms of the enhancement factor, as shown earlier in Figure 1. In that purpose, the maximum breakdown enhancement factor η_*Emax*_, that normalizes the highest *E*_*BD*_ result obtained for PI/SiO_2_ nanocomposite at 2 wt % to the one of neat PI, has been calculated following eq 2:

$$\eta_{Emax}=\left(\frac{E_{\mathrm{BD PI}/\mathrm{Si}O_{2}}}{E_{\mathrm{BD PI}}}-1\right)\times 100$$ 2

Figure 12 compares the maximum breakdown field strength *E*_*BDmax*_ versus the enhancement factor η_*Emax*_ obtained for PI/SiO_2_ nanocomposite at 2 wt % (both in peak and rms values) with respect to the state-of-the-art for the main documented PI-based nanocomposites. The graph also shows the breakdown field for pure PI in each case. All these extracted data can also be found in Table S3. As introduced earlier at the top of this paper, the state-of-the-art of polyimide-based nanocomposites has been for a long time capped below *E*_*BD*_ < 650 V/μm. Moreover, most of the enhancements reported were usually ranging between η_*Emax*_ ∼ [+10%; + 40%] with very rare examples beyond. Our results report here a 2-fold spectacular enhancement that significantly pushes forward the state-of-the-art boundaries. First, *E*_*BDmax*_ has been increased to reach for the first time ever the *ultrahigh breakdown field strength* for polyimide-based nanocomposite films with a new world record established as high as ∼1000 V/μm. On the other hand, with an enhancement factor η_*Emax*_ = +67.8%, it places also this PI/SiO_2_ nanocomposite film in the highest range in terms of percentage enhancement of the breakdown field. Such performances particularly highlight the key role of the nanofiller functionalization and dispersion optimization that have been developed here.

**Figure 12:**
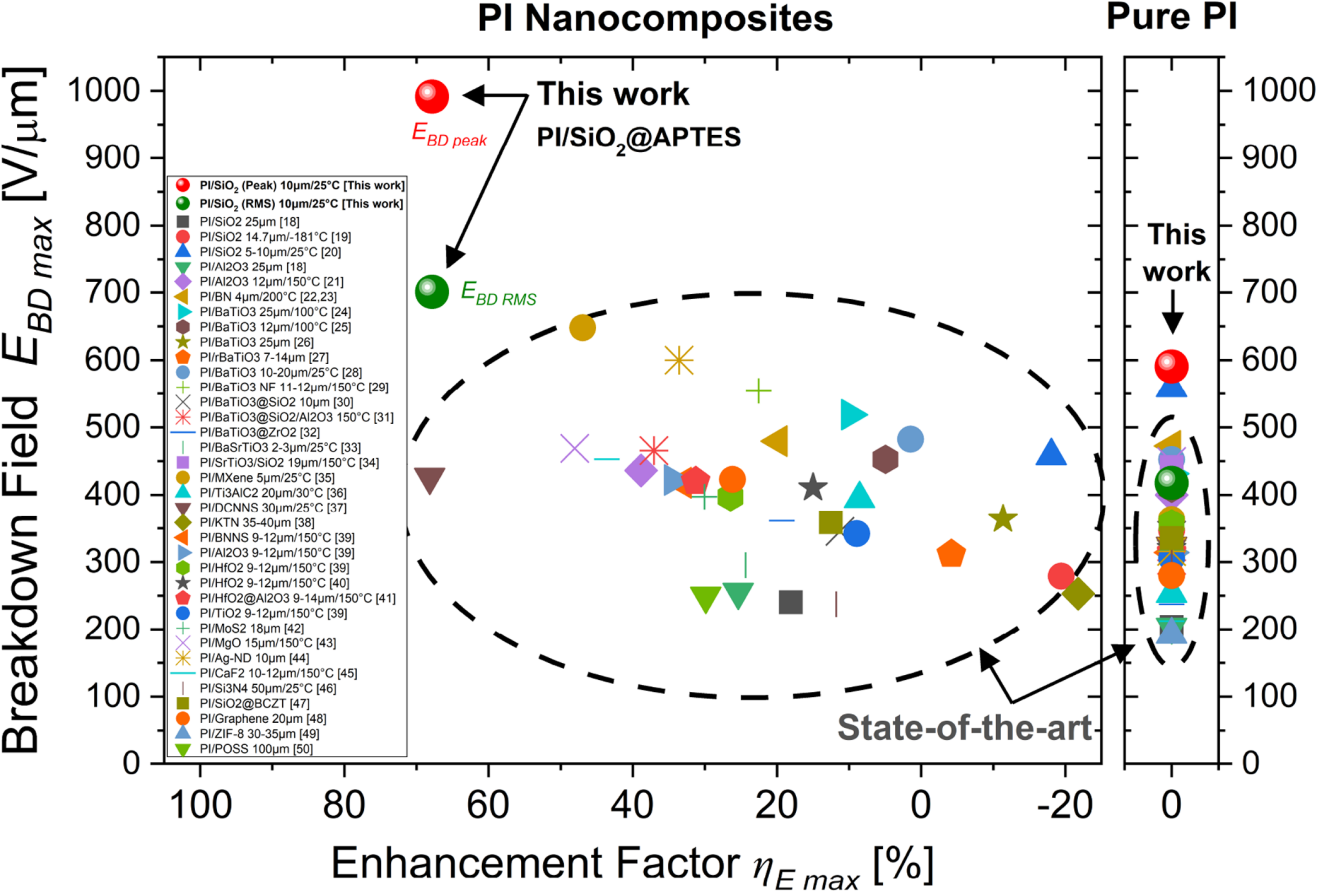
Comparison of the maximum breakdown strength *E*_*BD max*_ and the breakdown enhancement factor η_*E max*_ of PI/SiO_2_@APTES nanocomposite film (both peak and RMS values) with respect to the main state-of-the-art PI-based nanocomposites with various nanoparticles (data from refs 18–50 and in Table S3). Film thickness and measurement temperature are indicated in the caption when available from the literature. Breakdown fields are expressed in DC or peak values for the literature data for ease of comparison. The right plot shows the breakdown strength of pure PI films for each literature reference.

Figure 13a,b presents a further analysis of the results replotted as a function of the thickness of the polyimide-based nanocomposite films. There is a clear trend in the literature that shows the thickness dependence of the breakdown field *E*_*BDmax*_, which follows an inverse power law (Figure 13a). Interestingly, the thinner the PI-based nanocomposite films, the higher the dielectric breakdown strength, with maxima obtained for thicknesses for 10 μm and below. In the present case of the PI/SiO_2_ nanocomposite films of 10 μm, their higher breakdown performances are significantly ahead of the current state-of-the-art and open new paths for improving the insulation properties in such a thickness range suitable for capacitors, galvanic isolation, or passivation applications. In addition, the state-of-the-art for the enhancement factor η_*Emax*_ looks more randomly scattered versus the film thickness and no clear tendency emerges (Figure 13b). However, one can note that the highest improvements reported were corresponding to PI-based nanocomposites >20 μm and in which the pure PI matrix had an initial lower breakdown strength (<280 V/μm). On the contrary, for film thicknesses below 20 μm, our PI/SiO_2_ nanocomposite film presents the highest enhancement factor of the breakdown field and emphasizes again the effectiveness of our fabrication method.

**Figure 13:**
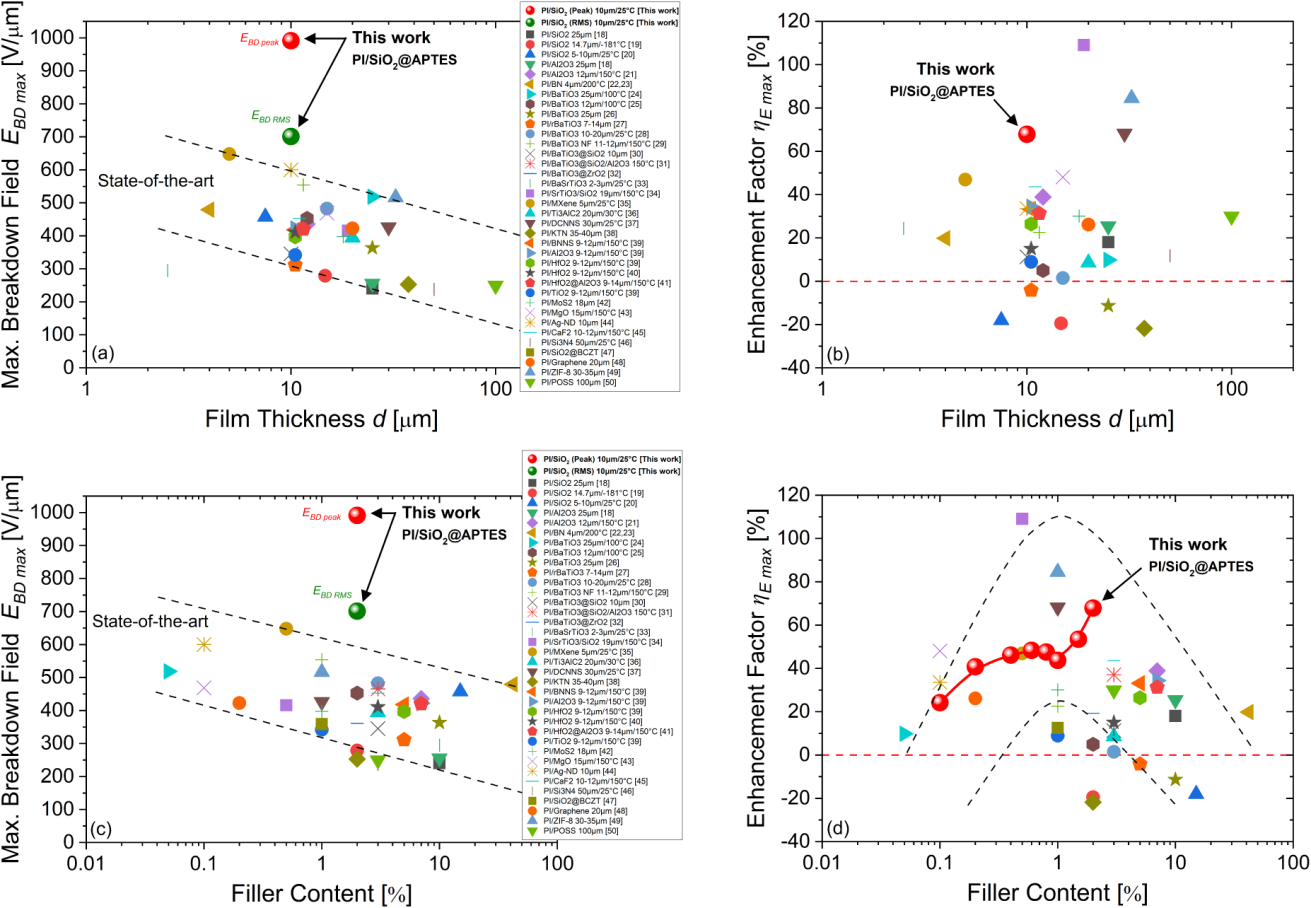
Thickness dependence (a, b) and filler content dependence (c, d) of the maximum breakdown strength *E*_*BD max*_ and the breakdown enhancement factor η_*E max*_ of the PI/SiO_2_@APTES nanocomposite film with respect to the state-of-the-art of various PI-based nanocomposites. Film thickness and measurement temperature are indicated in the caption when available from the literature. Breakdown fields are expressed in DC or peak values for the literature data for ease of comparison.

Alternatively, Figure 13c,d presents the main results replotted now as a function of the filler content. In this representation, the polyimide-based nanocomposite films data can appear differently ranked. Regarding the dielectric strength, *E*_*BDmax*_ shows an overall decrease with increasing nanoparticle concentration, as is often reported (Figure 13c). Despite this expected tendency, in the current PI/SiO_2_ nanocomposite film, the possibility to significantly improve the breakdown field even at very low filler content was obtained considering the fact that the nanoparticles are efficiently dispersed within the PI matrix. This likely means that the combined effects of the optimized ligand grafting on the nanoparticle surface with their good mechanical dispersion has enabled to massively tune the interfacial properties between SiO_2_ and the PI chains as soon as these small contents of nanofillers. On the other hand, the enhancement factor η_*Emax*_ appears now with a fair filler content dependence (Figure 13d). Then, the enhancement factor η_*Emax*_ exhibits a maximum that could empirically be located around the unit of percent of the filler content range. Within this trend, the current PI/SiO_2_ nanocomposite films fit well and stand in the highest range of percentile improvements. Moreover, they even show a continuous enhancement up to 2 wt %.

Finally, Figure 14 summarizes the improvements of all the different electrical and dielectric properties investigated here between the pure PI and the PI/SiO_2_ nanocomposite film (2 wt %) and plotted in the form of radar areas.

**Figure 14:**
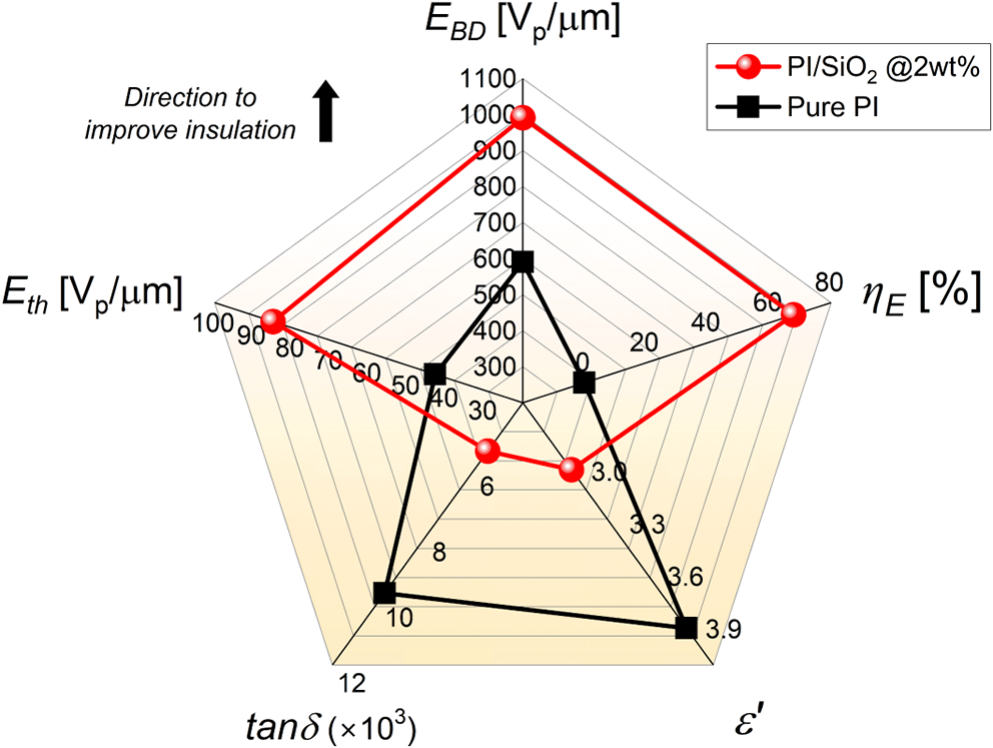
Radar areas for comparing the main dielectric and electrical properties obtained in pure polyimide and 2 wt % polyimide/SiO_2_ nanocomposite at 50 Hz and room temperature. The upward arrow indicates the direction of change expected to achieve an improved insulation.

This comparison clearly shows how this work further expanded the electrical performance boundaries of polyimide films by tailoring them with SiO_2_ nanoparticles. Therefore, all the main electrical properties and characteristics have been enhanced, shifting them toward values that will allow for the improvement of the insulation rating. Thus, in the case of the PI/SiO_2_ nanocomposite, both the permittivity *ε’* and loss factor *tanδ* have been mitigated, while the nonlinear threshold field *E*_*th*_, the breakdown field *E*_*BD*_ and the enhancement factor η_*E*_ have been significantly increased. Such exceptional insulation performances make these PI/SiO_2_ nanocomposite films excellent candidates for integration in future generations of advanced insulating films for higher voltage isolation and capacitive energy storage applications.

## 4 Conclusions

This work reports the enhancement of the dielectric breakdown strength of polyimide-based nanocomposite films tailored with silica (SiO_2_) nanoparticles. A successful functionalization of SiO_2_ nanoparticles using a 3-aminopropyltriethoxysilane (APTES) ligand is reported. Chemical characterizations (FTIR, XPS, ζ-potential) have confirmed the effective grafting of single-layer ligand coverage onto the nanoparticle surface and their optimal colloidal stability. The optimized nanoparticle functionalization conditions promote their homogeneous dispersion within the PI matrix, as shown through AFM surface topography and cross-sectional FIB/SEM imaging. APTES-functionalized PI/SiO_2_ nanocomposite films with various filler contents have shown improved electrical and dielectric properties with lower permittivity and loss factor, and a shift of the nonlinear conductivity threshold to higher electric field. Such improvements are related to more efficient charge trapping effects, likely in the interphase regions. Moreover, a massive enhancement of the breakdown field strength of ∼68% for the PI/SiO_2_ nanocomposite compared to pure PI films has been achieved. This study highlights for the very first time the path to achieving revolutionary ultrahigh breakdown field strength for polyimide-based nanocomposite films with a new state-of-the-art achievement of *E*_*BD*_ ∼ 1000 V/μm.

## Supplementary Material



## Data Availability

The data that support the findings of this study are available from the corresponding author upon reasonable request.

## Notes

Breakdown voltage setup details (Figure S1); dielectric permittivity vs frequency of the nanocomposites (Figure S2); and state-of-the-art database used for PI nanocomposite breakdown comparison (Table S3) (PDF)
