# Supplementary material for: Polyimide-Based Nanocomposites with Ultra-High Dielectric Breakdown Strength: A Review and New Record
Source: ACS Appl Electron Mater. 2025 Oct 21;7(21):9729–42. doi: 10.1021/acsaelm.5c01479 (PMC12856994; doi:10.1021/acsaelm.5c01479)
Supplement: Supplementary file 1 [file el5c01479_si_001.pdf]

# - Supporting Information -

Polyimide-based nanocomposites with ultra-high dielectric breakdown strength: review and new record

Sombel Diaham[1,*], Imadeddine Benfridja[1,2,3] and Tadhg Kennedy[2,3]
[1]*Université de Toulouse, Toulouse INP, CNRS, LAPLACE, Toulouse, 31062, France.*
[2]*Department of Chemical Sciences, University of Limerick, Limerick, V94 T9PX, Ireland.*
[3]*Bernal Institute, University of Limerick, Limerick, V94 T9PX, Ireland.*

Corresponding author: sombel.diaham@laplace.univ-tlse.fr

S1: AC breakdown voltage characterization

Breakdown testing was carried out using a high voltage probe station configured to observe real-time voltage and current waveforms and the ability to record the failure location with a CCD camera (Fig. S1). For AC breakdown voltage (V_{BR}) measurements, the test structures were biased by applying the high voltage on top electrode whilst the bottom metal layer was grounded using contact needles controlled with micro-positioners. The testing was carried out by amplifying a low amplitude, 50 Hz, AC sinewave voltage coming from a waveform generator using an HV amplifier (gain: ×3000, slew rate: >500 V/µs, bandwidth: >10 kHz, distortion: <2%). The amplifier output voltage, $V_m(t)$, was applied to the sample and detected on an oscilloscope after attenuation via a HV probe (1/1000). The total current, $i(t)$, flowing across the sample was collected on the oscilloscope through a clamp-on high-frequency current transformer (HFCT, bandwidth 120 MHz, 10 µA accuracy). The HV voltage ramp rate was 1 kV_p/s following the recommended test duration specified in the international ASTM D149-20 standard. Fig. S1 shows a sketch of the experimental breakdown test bench.

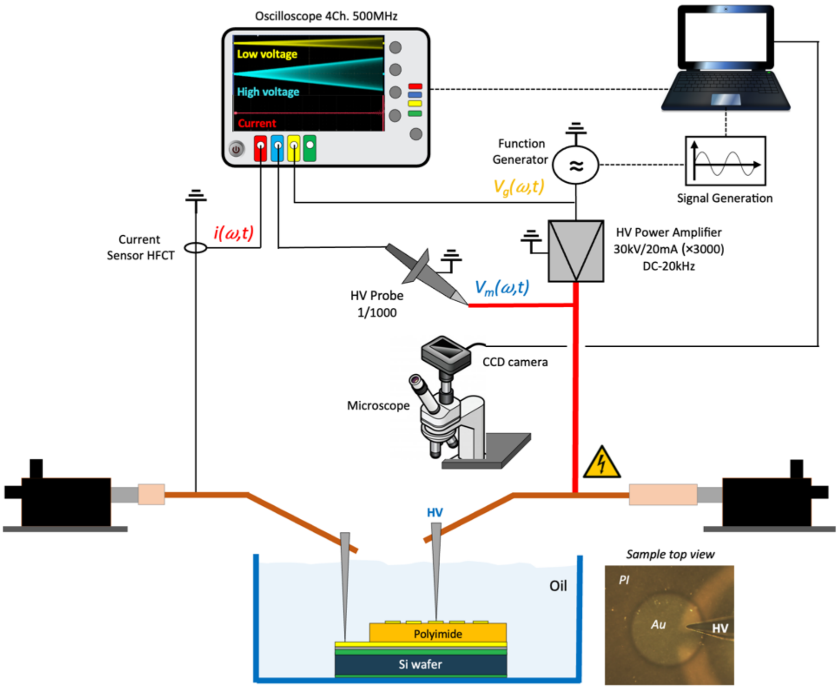

Figure **S1**. Experimental setup for AC breakdown voltage testing with simultaneously current-voltage pre-breakdown waveform probing.

S2: Permittivity

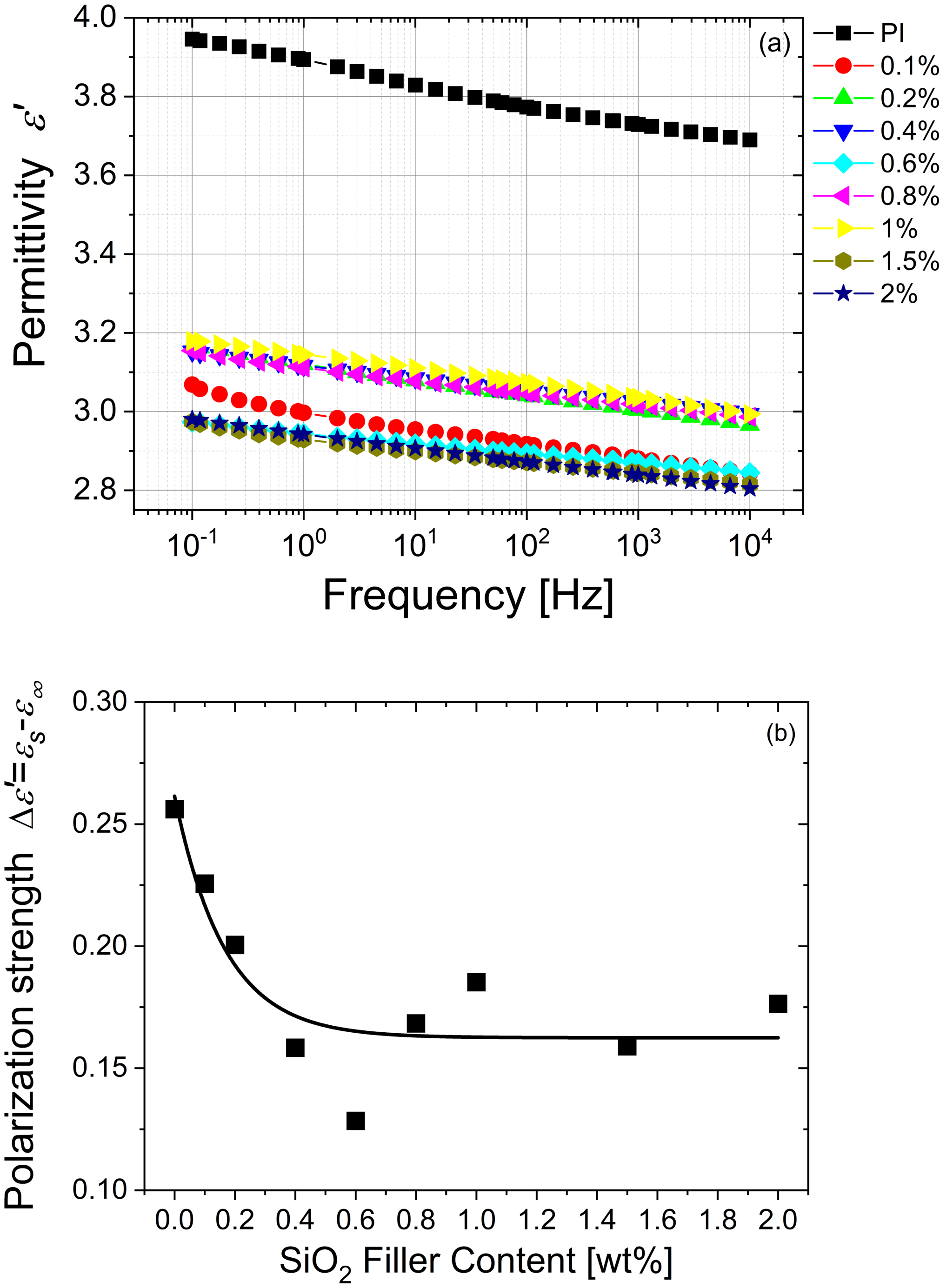

Figure **S2**. Frequency-dependence of the permittivity *ε'* between 100 mHz and 10 kHz (a) and the polarization difference *Δε'* throughout this frequency range (b) in PI/SiO_2@APTES nanocomposite films as a function of the filler content.

S3: State-of-the-art available data extracted from literature

Table **S3**. Comparison of the maximum breakdown strength $E_{BD\ max}$ and enhancement factor $\eta_{E\ max}$ of PI/SiO_2@APTES with the main state-of-the-art polyimide-based nanocomposites with various nanoparticles. References are numbered as shown in our core paper. Empty boxes correspond to unavailable information from these references.

N°	Polyimide/nanofiller	Content range	NP size [nm]	Film thickness [µm]	Temperature [°C]	Max. breakdown strength $E_{BD\ max}$ [V/µm]	Maximum enhancement factor $\eta_{E\ max}$	Ref.
1	PI/SiO_2	1-10wt%		25		170 (AC)	+18%	[18]
2	PI/SiO_2	2-6wt%	100-300	14.7	-181 (92K)	279	-19.4%	[19]
3	PI/SiO_2	15-30vol%	29	5-10	25	457.8	-18%	[20]
4	PI/Al_2O_3	1-10wt%		25		180.5	+25.4%	[18]
5	PI/Al_2O_3	1-7vol%	30	12	150	436	+38.9%	[21]
6,7	PI/BN	1.6-42vol%	35	4	200	479.3	+19.8%	[22,23]
8	PI/$BaTiO_3$	0.05-50wt%	100	25	100	520	+9.8%	[24]
9	PI/$BaTiO_3$	2-8vol%	30-60	12	100	453	+5%	[25]
10	PI/$BaTiO_3$	10-70wt%	100	24-26		363.2	-11.3%	[26]
11	PI/r$BaTiO_3$	5-50wt%	200-500	7-14		311.5	-4.2%	[27]
12	PI/$BaTiO_3$	1-9 vol%	140	10-20	25	482.5	+1.46%	[28]
13	PI/$BaTiO_3$ NF	1-9 vol%	<500	11-12	100-200	554	+22.5	[29]
14	PI/$BaTiO_3$@SiO_2	1-5vol%	300	10		346	+12%	[30]
15	PI/$BaTiO_3$@SiO_2/Al_2O_3	3vol%	200/10/250		150	465	+38%	[31]
16	PI/$BaTiO_3$@ZrO_2	1-5vol%	400/30			361	+19.1	[32]
17	PI/$BaSrTiO_3$	5-18vol%	9.5	2-3	25	296	+24%	[33]
18	PI/$SrTiO_3$/SiO_2	0.3-2 vol%	100/20	18-20	150	415.7	+109%	[34]
19	PI/MXene	0.5-3wt%		5	25	648	+46.9%	[35]
20	PI/Ti_3AlC_2	1-7wt%	300-400	20	30	394.5	+8.8%	[36]
21	PI/DCNNS	0.25-1wt%		30	25	300 (AC)	+67.6%	[37]
22	PI/KTN	2-8wt%	60-100	35-40		253	-21.8%	[38]
23	PI/BNNS	2-6vol%		9-12	150	418	+33.1%	[39]
24	PI/Al_2O_3	1-9vol%	30	9-12	150	422	+34.4%	[39]
25	PI/HfO_2	1-9vol%	50	9-12	150	397	+26.4%	[39]
26	PI/HfO_2	1-9vol%	60-80	9-12	150	407	+14%	[40]
27	PI/HfO_2@Al_2O_3	3wt%	50-80/8	9-14	150	420.3	+31.3	[41]
28	PI/TiO_2	1-9vol%	30	9-12	150	342	+8.9%	[39]
29	PI/MoS_2	0.5-2vol%		18		395	+29.5%	[42]
30	PI/MgO	0.05-0.3vol%	30	15	150	469	+47.9%	[43]
31	PI/Ag-ND	0.1-1vol%	5-15	10		600.1	+33.4%	[44]
32	PI/CaF_2	1-7vol%	100	10-12	150	455.4	+44.6%	[45]
33	PI/Si_3N_4	1-7wt%	10-30	50	25	237.4	+11.8%	[46]
34	PI/BCZT@SiO_2	1-7vol%	570/20			358.8	+12.5%	[47]
35	PI/Graphene	0.1-1.5vol%		20		299 (1kHz)	+26.1%	[48]
36	PI/ZIF-8	1-5wt%		30-35		516.3	+84.5%	[49]
37	PI/POSS	3wt%		100		249.5	+29.9%	[50]
38	PI/SiO_2	0.1-2wt%	18	9.4-10.4	25	991.4 (peak) 701 (rms)	+67.8%	This work

Note: These data have been extracted from each paper either from the text or by image vectorization when not readily available.
